# The Anatomic Landmark Approach to Extratemporal Facial Nerve Repair in Facial Trauma

**DOI:** 10.7759/cureus.22787

**Published:** 2022-03-03

**Authors:** An Q Lam, Thuy Tran Phan Chung, Luan Tran Viet, Hung Do Quang, Duong Tran Van, Albert J Fox

**Affiliations:** 1 Department of Plastic and Aesthetic Surgery, Cho Ray Hospital, Ho Chi Minh City, VNM; 2 Department of Otolaryngology, Vietnamese National University School of Medicine, Ho Chi Minh City, VNM; 3 Department of Otolaryngology, Pham Ngoc Thach University of Medicine, Ho Chi Minh City, VNM; 4 ENT - Facial Plastic Surgery, Albert Fox Facial Plastic Surgery Center, Dartmouth, USA; 5 ENT - Facial Plastic Surgery, Southcoast Hospital Group, New Bedford, USA

**Keywords:** maxillofacial injuries, facial trauma, facial nerve surgery, facial nerve injury, facial nerve repair, maxillofacial trauma, facial nerve paralysis

## Abstract

Objective

In this study, we aimed to examine the topical anatomic landmarks of the facial nerve (facial nerve areas) and their application in cases of extratemporal facial nerve injury in maxillofacial trauma.

Materials and methods

We analyzed 25 maxillofacial trauma patients with facial paralysis who underwent facial nerve reanimation surgery at the Ho Chi Minh City National Hospital of Odonto-Stomatology. The characteristics of each trauma case, including the mechanism of injury, the length of the facial injury, and the location/position of injury, were recorded. The association of the injured nerves with the trauma characteristics and the external landmarks of the facial danger zones was analyzed.

Results

The buccal branches had the highest rate of paralysis (22/25 cases), followed by zygomatic branches (15/25), frontal branches (11/25), marginal branches (6/25), and the main trunk (1/25). There were four areas related to the external facial nerve landmarks (facial nerve areas) that helped us find the affected nerves: wounds in Area 1 resulted in frontal branch paralysis in five out of eight cases (62.5%); wounds in Area 2 resulted in zygomatic branch paralysis in 8/13 cases (61.5%) and buccal branch paralysis in 12/12 cases (100%); wounds in Area 3 resulted in marginal branch paralysis in 5/10 cases (50%); and wounds in Area 4 alone resulted in main trunk paralysis in one out of four cases or at least two main branches in three out of four cases.

Conclusion

Extratemporal facial paralysis after facial trauma can be complex and highly variable, leading to difficulty in finding and repairing facial nerves. Thorough clinical examination and evaluation of trauma characteristics can aid in the identification of facial paralysis and repair. Mapping facial wounds using the four anatomic surface landmarks (Areas 1-4 as outlined in this research) helped us anticipate which branches might be traumatized and estimate the position of the distal and proximal endings to repair the nerves in all cases.

## Introduction

In developing countries, injuries caused by traffic and labor accidents and assault are a significant concern. Maxillofacial trauma accounts for 16% of major trauma injuries [1]. Maxillofacial trauma includes injuries to the soft tissue, facial bone fractures, and facial paralysis. Without appropriate treatment, patients with facial paralysis can suffer from severe sequela resulting in cosmetic issues as well as affecting functional aspects of daily lives and social communication [2].

Microsurgical techniques have enabled surgeons to re-anastomose the facial nerve. However, in post-traumatic cases, the surgeon needs to find the proximal and distal end of each injured branch to perform end-to-end re-anastomosis or use a nerve graft to maximize the surgical outcome [1]. Facial nerve branching is complex and many of its branches are small and difficult to find and dissect [2]. Furthermore, in traumatic cases, the anatomy of the structures can be altered due to soft tissue damage, bone fractures, and scar contracture. Consequently, exploring the injured facial nerve, and identifying the proximal and distal ends is difficult. Understanding the surgical anatomic landmarks of the facial nerve and their relationship with surrounding structures can aid the surgeon in the repair of the injured facial nerve [3].

## Materials and methods

This study involved 25 patients who suffered extratemporal bone facial nerve injuries caused by traffic and labor accidents and assault; they were hospitalized at the Ho Chi Minh City National Hospital of Odonto-Stomatology during the period spanning December 2016 to December 2018. These patients presented with facial wounds and signs of facial paralysis. We recorded the trauma history, as well as the time, causes, and mechanisms of injury. We measured the lengths of the wound in each of the four zones mentioned above. Each wound was then surgically explored to find the traumatized facial nerve together with both proximal and distal ends. If the distance between the injured proximal and distal ends did not exceed 2 cm, we dissected the nerve on both sides and performed an end-to-end re-anastomosis. Otherwise, we used the sural nerve as a graft to restore the nerve continuity. We applied our knowledge of the external facial nerve anatomic landmarks (facial nerve areas) as described below to assess the areas of facial nerve injury.

**Figure 1:**
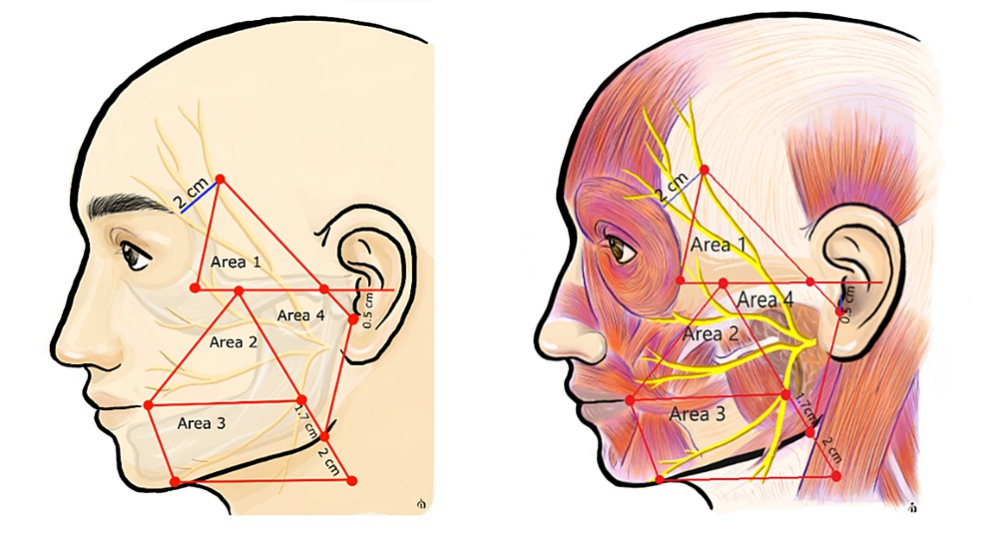
External landmarks of the facial nerve with four Areas

The anatomic landmarks of the facial nerve were described by Seckel et al. in 1994 and were divided into danger zones to prevent nerve injury in facial plastic surgery [4]. Later on, Rohrich et al. outlined the branching and the relationship between the facial nerve and the surrounding tissue, such as bone structures, blood vessels, and fixed points [5]. Baur et al. delineated the relationship between the marginal mandibular nerve and the inferior border of the mandible [6]. Based on the studies mentioned above, we divided our facial trauma patients' faces into four areas as described below to evaluate the facial nerve (Figure 1).

Area 1: a triangle formed by drawing the following intersecting lines [4]

- A line starting 0.5 cm below the tragus to a point 2 cm above the lateral eyebrow

- A line along the zygomatic bone to the lateral orbital rim

- A line from the lateral orbital rim to the point 2 cm above the lateral eyebrow

This zone contains the frontal branches above the zygomatic bone before entering the frontalis and orbicularis oculi muscles.

Area 2: a triangle formed by intersecting lines at the following three points

- Malar eminence [7]

- Oral commissure [7]

- A point 1.7 cm above the mandibular angle [6]

This zone contains the zygomatic and buccal branches of the facial nerve.

Area 3: a quadrangle outlined by four points [7]

- Oral commissure

- A point directly inferior to the oral commissure on the lower border of the mandible

- A point 1.7 cm above the angle of the mandible

- A point 2 cm below the mandibular angle, in the line from malar eminence to the mandibular angle

This zone contains the marginal branch and a part of the cervical branches.

Area 4: a quadrangle outlined by four points

- Malar eminence

- Mandibular angle

- A point 0.5 cm below the tragus

- Intersection point of the zygomatic line and the line starting 0.5 cm below the tragus to a point 2 cm above the lateral eyebrow 

This area contains the facial nerve in the parotid gland, including the main trunk and branches. This area lies posterior to Areas 2 and 3.

## Results

In this study, 25 maxillofacial trauma patients with facial paralysis (four females and 21 males) with an average age of 35.28 years (range: 20-58 years) were analyzed. All 25 cases had been transferred from outside facilities after their facial lacerations had been closed; 10/25 (40%) cases with facial paralysis were not primarily diagnosed. The median time from trauma to facial nerve repair was 17 days (range: 11-26 days). Of note, 22/25 patients required concurrent facial nerve, facial fracture, and Stenson’s duct repair; 3/25 required facial nerve repair only.

Among the patients, the etiology of trauma was as follows - assault (48%), motorbike accident (32%), and work accident (20%). Of the causes of trauma, the predominant mechanism of injury was sharp injury (64%), including those caused by a knife (eight cases), glass (five cases), saw (two cases), and metal weapon (one case). The details of each injured facial nerve branch (e.g., frontal, zygomatic) and paralysis of smaller branches from those respective nerves are outlined in Table 1.

**Table 1:** Number of cases of facial nerve injury and small branch injury

Injured nerve	Number of small branches	Cases	Total
Frontal branch	1	10	11
	2	1	
Zygomatic branch	1	3	15
	2	12	
Buccal branches	1	12	22
	2	8	
	3	1	
	4	1	
Marginal branches	1	4	6
	2	2	
Cervical branches	0	0	0
Main trunk	0	1	1

In our cohort, the buccal and zygomatic branches had the highest injury rates, followed by frontal branches and marginal branches. There was only one case with main trunk trauma, and there was no case of cervical branch injury.

**Figure 2:**
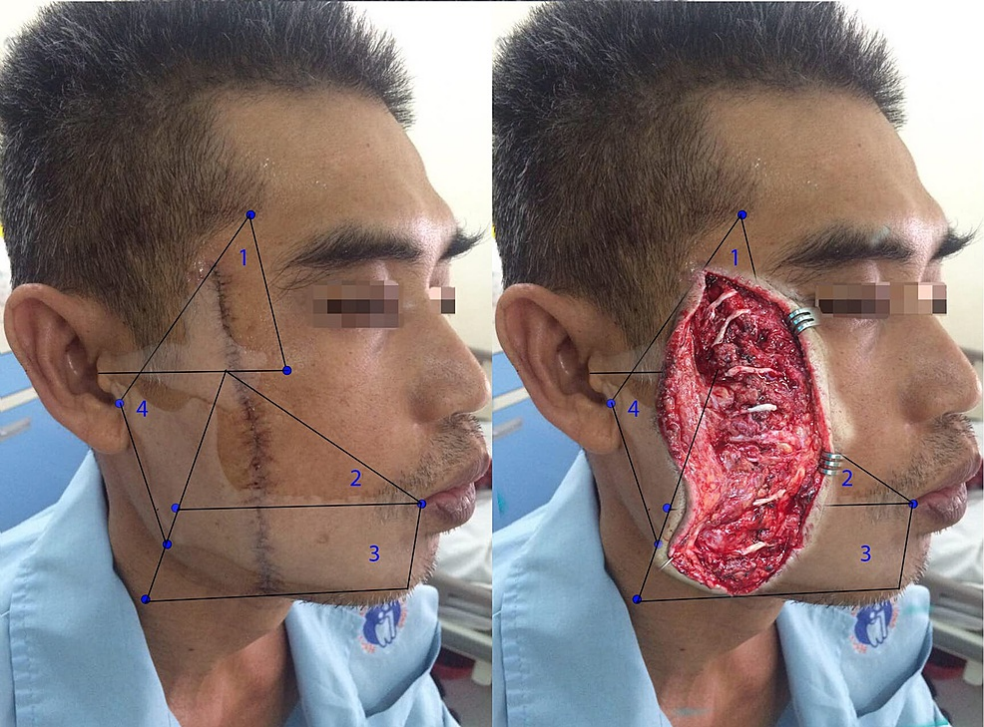
Facial trauma patient with the wound affecting Areas 1, 2, and 3

There were no cases where the wound affected all four areas mentioned above. The wounds involving one area, two areas, and three areas were 36%, 36%, and 28%, respectively. The majority of patients had wounds in Area 2 (17, 68%); The number of cases with wounds in Area 3, Area 1, and Area 4 was 12 (48%), 10 (40%), and nine (36%), respectively. Figure 2 shows a patient with injury affecting Areas 1, 2, and 3.

The relationship of the total length of the laceration with the length of the laceration, specifically within Areas 1-4, and the number of paralyzed facial nerve branches are presented in Table 2.

**Table 2:** The relationship between the number of main branch paralysis and the length of laceration *ANOVA test ANOVA: analysis of variance; SD: standard deviation

Number of branches paralyzed	N	Length of laceration (cm), mean ±SD	P1	Length of laceration in Areas 1-4, (cm), mean ±SD	P2
1 branch	7	7.4 ±3.3	0.081*	4.9 ±1.8	0.018*
2 branches	7	9.4 ±3.5		7.8 ±3.4	
3 branches	7	8.4 ±4.5		6.9 ±3.2	
4 branches	3	15.1 ±6.1		11.7 ±1.6	
Total	24				

The average entire length of laceration and the length of laceration in the four Areas were 9.1 ±4.5 cm and 7.1 ±3.3 cm, respectively. The total number of facial nerve branches affected in relation to the length of the laceration within the four Areas was statistically significant, while there was no relationship between the entire length of laceration and the number of facial nerve branches affected. The relationship of the traumatized facial nerve branches with their respective Areas of involvement is outlined in Table 3.

**Table 3:** Relationship between the traumatized branches and wounds in the four Areas

Facial nerve branch	Area	Number of cases	Total
Frontal	Area 1	5	11
	Area 4	5	
	Areas 1 and 4	1	
Zygomatic	Area 2	8	15
	Area 4	3	
	Areas 2 and 4	4	
Buccal	Area 2	14	22
	Area 4	4	
	Areas 2 and 4	4	
Marginal	Area 3	5	6
	Area 4	1	
	Areas 3 and 4	0	
Main trunk	Area 4	1	1

Frontal branch paralysis occurred only in cases where Area 1 and/or Area 4 were affected. Zygomatic and buccal branch paralysis occurred only in cases where Area 2 and/or Area 4 were affected. Marginal branch paralysis occurred only in cases where Area 3 and/or Area 4 were affected. Main trunk paralysis occurred only in cases where Area 4 was affected. Without having a wound in Area 4, the relationship of Area 1 with frontal branches paralysis, Area 2 with zygomatic and buccal branches, and Area 3 with the marginal branches were as outlined in Table 4.

**Table 4:** Relationship between nerve branch paralysis and Areas 1, 2, and 3 when Area 4 was intact

Types of branches		Area		Number of cases
		Area 1		Total
		Not affected	Affected	
Frontal	Intact	8	3	11
	Trauma	0	5	5
Total		8	8	16
P=0.026 (Fisher's exact test)				
		Area 2		Total
		Not affected	Affected	
Zygomatic	Intact	2	6	8
	Trauma	0	8	8
Total		2	14	16
P=0.46 (Fisher's exact test)				
		Not affected	Affected	
Buccal	Intact	2	0	2
	Trauma	0	14	14
Total		2	14	16
P=0.008 (Fisher's exact test)				
		Area 3		Total
		Not affected	Affected	
Marginal	Intact	6	5	11
	Trauma	0	5	5
Total		6	10	16
P=0.09 (Fisher’s exact test)				

Wounds in Area 1 led to frontal branch paralysis in 62.5% of cases (five out of eight cases). Wounds in Area 2 caused zygomatic branch paralysis in 57.1% of cases (8/14 cases) and buccal branch paralysis in 100% of cases (14/14 cases). Wounds in Area 3 caused marginal branch paralysis in 50% of cases (5/10 cases). There were five cases with the wound in Area 4 associated with other areas and four cases with a wound only in Area 4. Among the cases with the wound only in Area 4, there was one case with main trunk paralysis and three cases with at least two main branches with paralysis.

## Discussion

Facial nerve paralysis can be divided into five major types based on etiologies: idiopathic, infectious, neoplastic, neurologic, and traumatic. Bleicher et al. [8] found that traumatic causes, mostly due to temporal bone fracture, were associated with the lowest incidence of facial nerve paralysis (8.2%). In our study, 40% of cases (10/25) with extratemporal facial paralysis were missed in primary diagnosis. The missed facial paralysis diagnosis was likely due to soft tissue injury, facial fractures, and edema distorting the face and concealing facial paralysis symptoms.

All 25 cases in our study were primarily sutured in other hospitals before being transferred to the Ho Chi Minh City National Hospital of Odonto-Stomatology for advanced treatment. Of note, 23/25 cases (92%) underwent facial nerve repair more than seven days after trauma; most repairs occurred 11-26 days post-trauma (one case was operated on the second day, and one case was operated on the sixth day). The reason for delayed repair could be attributed to the following factors: patients with facial trauma usually present with multiple organ injuries or other life-threatening conditions, requiring critical management, thereby resulting in delayed facial palsy diagnosis and treatment; facial bone fractures and tissue injuries in facial trauma concealed the identification of facial paralysis; inexperienced physicians would repair the laceration on the face without confirming the status of the facial nerve. If left untreated, facial paralysis will affect the aesthetics, function, and patient's quality of life [9,10].

Theoretically, the sooner the facial nerve is repaired, the better the outcome of the surgery [1]. In acute trauma, nerve repair done within 72 hours has the highest rate of recovery because Wallerian degeneration does not occur in such cases and the distal nerve endings can still be stimulated [11]. We used the Vari-Stim III Nerve Locator (Medtronic Xomed INC, Jacksonville, FL) to stimulate the mimetic muscle to find the distal nerve endings [12]. However, in our study, many of the nerve repair surgeries occurred seven days post-trauma, where Wallerian degeneration occurred. Due to a combination of Wallerian degeneration, scar contracture, and soft tissue and muscle trauma, the nerve locator did not help localize the traumatized nerves. The use of the external facial nerve landmarks (Areas 1-4) as a mapping technique may be helpful in such circumstances to find traumatized nerve endings.

In acute and sharp trauma usually caused by sharp objects like knives, swords, or glass in the assault, the wound is well demarcated with minimal distortion. Therefore, dissection for traumatized nerves was feasible. However, in late, blunt, and complicated trauma or facial bone fractures, finding the affected nerves was more difficult. The facial nerve was often displaced, distorted, and deformed, and we found the affected nerves based on the clinical features and the wound characteristics of the trauma. Clinical presentations of facial paralysis in the upper, middle, and lower third of the face were evaluated to anticipate which branches would be affected [3,13]. We localized the wounds and compared them with the four external landmarks (Areas 1-4) to improve the possibility of finding the proximal and distal nerve endings [13].

Wounds in Area 1

**Figure 3:**
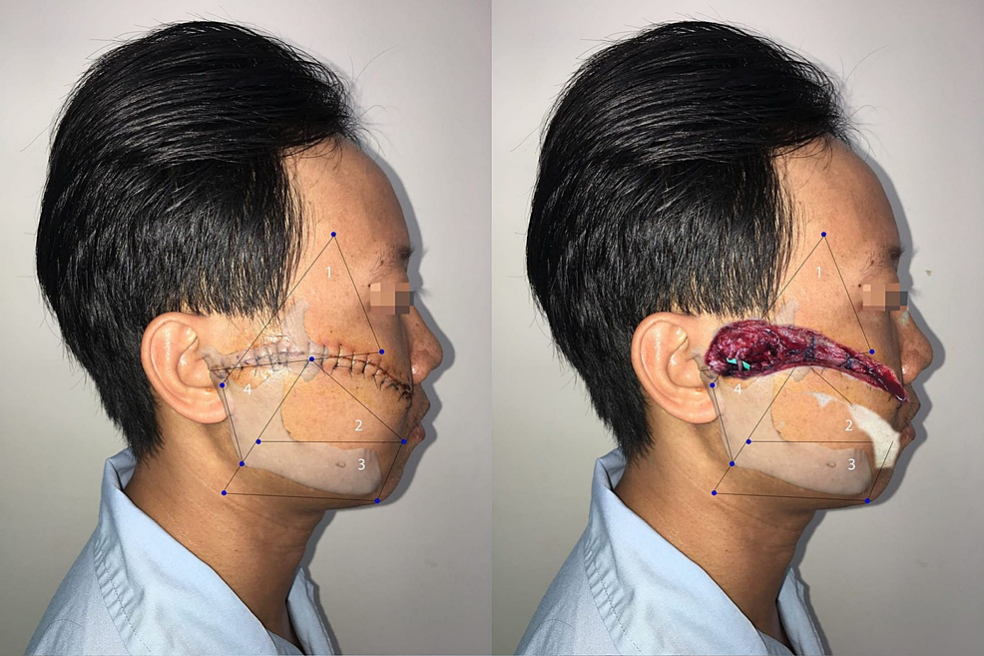
Patient with injury in Area 1 The patient had a 12.5-cm wound, but only 3.5 cm in Area 1. The patient had a frontal branch injury

This area usually contained most of the frontal branches [7]. The frontal nerve had one to six small branches and a few crossed the frontal branch of the superficial temporal artery [14]. In this area, Bonnecaze et al. recognized that the frontal branches crossed the inferior and superior border of the zygomatic bone at a distance from the anterior tragus of about 24.5 mm (range: 11.5-40 mm) and 28.25 mm (range: 19-40 mm), respectively [15]. Figure 3 shows a patient with injury in Area 1.

In patients with an intact Area 4, those having a wound in Area 1 had frontal branch paralysis in 62.5% of cases.

Wounds in Area 2

**Figure 4:**
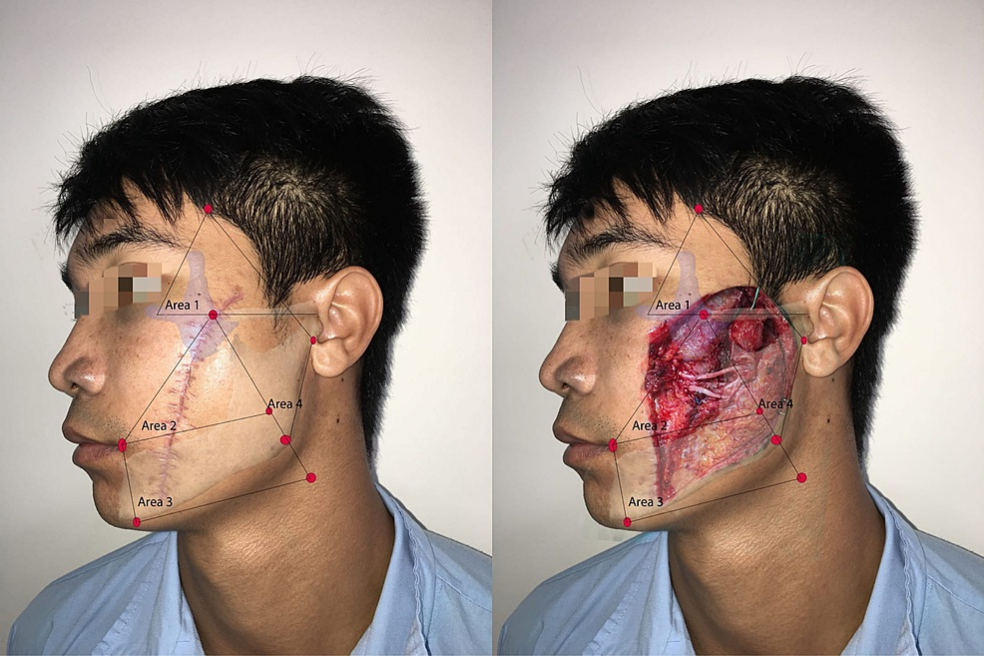
Patient with wounds in Areas 1, 2, and 3 In Area 2, the zygomatic and buccal branches were traumatized

This area contains the zygomatic and buccal branches after they exit the parotid gland. The zygomatic branch could have one to three branches, and the highest branch exits the parotid gland at a point with a distance to the tragus of 30.71 mm (range: 16.2-45.64 mm). The buccal branches and parotid duct cross each other within a semicircle with a 30-mm radius. The base (diameter) is parallel to a horizontal line passing the corner of the mouth and 12 mm above, and its center is located 53 mm lateral to it [16]. In our study, buccal and zygomatic branches had the highest rate of paralysis: 22/25 cases and 15/25 cases, respectively. The reason may be that the facial nerve branches in this area are more superficial after leaving the parotid gland. Figure 4 shows a patient with a wound involving Area 2.

With an intact Area 4, wounds in Area 2 resulted in 57.1% and 100% zygomatic and buccal branch paralysis, respectively.

Wounds in Area 3

**Figure 5:**
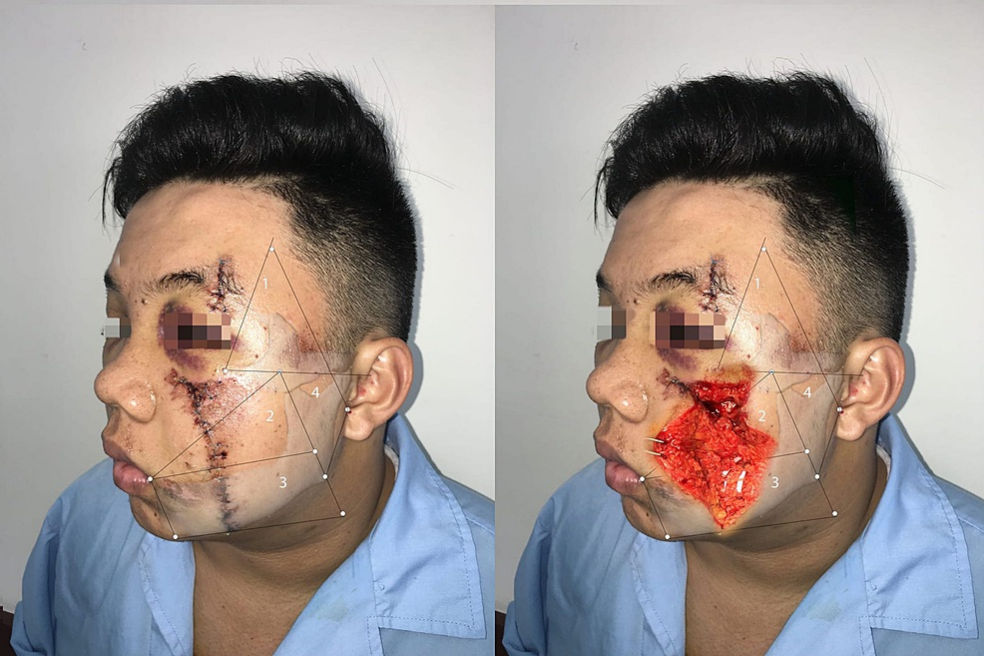
Patient with a wound in Areas 2 and 3 The patient had buccal and marginal branch paralysis

We were able to find the marginal and cervical branches in this area [17]. Marginal branches passed on an average of 0.75 mm below the gonion (range: -15 to 17 mm) [16]. According to Saylam et al. [18], marginal branches crossed the facial artery at the position above the lower border of the mandible in 80% of cases. In our study, marginal branch paralysis had the lowest incidence (6/25 cases), and no cervical branch paralysis was reported. Figure 5 shows a patient with sequela of a wound involving Area 3.

With an intact Area 4, 50% of cases having the wound in Area 3 had marginal paralysis in our study.

Wounds in Area 4

**Figure 6:**
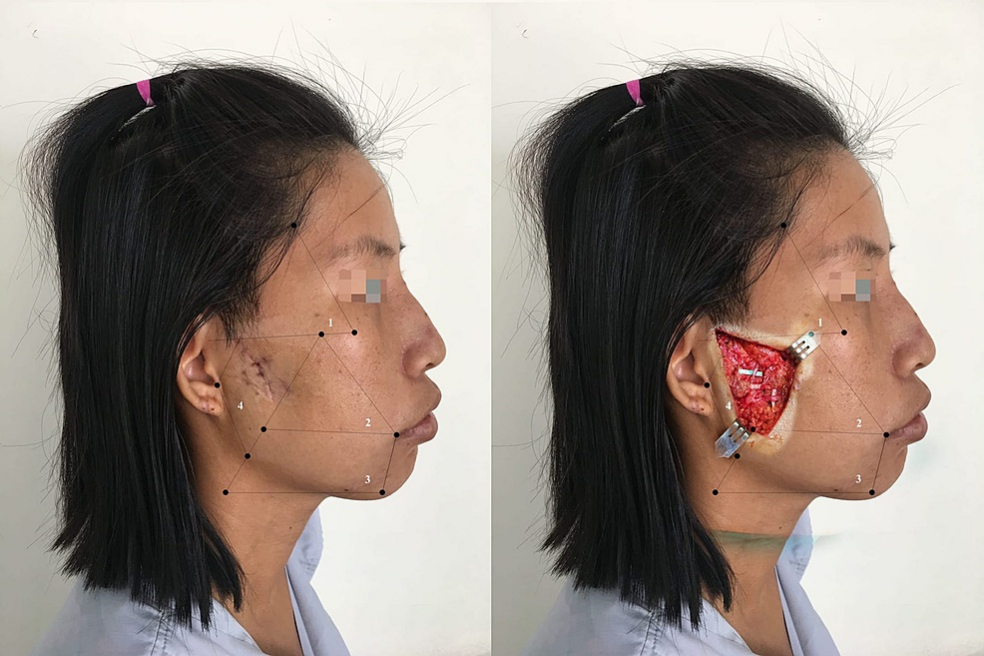
Patient with a wound in Area 4 The wound resulted in zygomatic, buccal, and marginal branch paralysis

This area contains the main trunk and the main branches of the facial nerve between the superficial and deep lobes of the parotid gland. The branching of the facial nerve in this area varied from left to right and from patient to patient. A wound in this area usually caused multiple main branch or main trunk paralysis (Figure 6).

In four cases with a wound only in Area 4, there was one case with main trunk paralysis and three cases with multiple main branch paralysis. In addition, the total number of facial nerve branches affected in relation to the total length of trauma in the four Areas was statistically significant, while there was no relationship between the total length of trauma and the number of facial nerve branches affected.

## Conclusions

Extratemporal facial paralysis after facial trauma was complicated and varied in our case series. Thorough clinical examination and evaluation of trauma characteristics in relationship with anatomic landmarks of the facial nerve can aid not only in the identification of facial paralysis but also in facial nerve repair surgery. Using the four anatomic surface landmarks (Areas 1-4 as outlined in this research) could help the surgeon anticipate which branches might be traumatized and estimate the position of the distal and proximal endings in facial nerve repair. Further studies comparing larger groups of patients with and without facial paralysis and injuries in Areas 1-4 may be helpful to determine the overall probability of facial nerve dysfunction associated with injuries in these specific zones. Also, future research comparing the effectiveness of utilizing the anatomic landmarks of the four areas described in this study with standard techniques using facial electromyography/nerve stimulator in finding injured nerves may be helpful in validating the efficacy of this technique in the management of traumatic facial nerve injury repair.
